# Association of *677 C>T (rs1801133)* and *1298 A>C (rs1801131)* Polymorphisms in the *MTHFR* Gene and Breast Cancer Susceptibility: A Meta-Analysis Based on 57 Individual Studies

**DOI:** 10.1371/journal.pone.0071290

**Published:** 2014-06-19

**Authors:** Kai Li, Wusheng Li, Xi Dong

**Affiliations:** Department of Oncology, Shengjing Hospital of China Medical University, Shenyang, Liaoning, P. R. China; Ohio State University Medical Center, United States of America

## Abstract

**Objective:**

The *677 C>T* and *1298 A>C* polymorphisms of methylenetetrahydrofolate reductase (*MTHFR*) gene have been widely reported and considered to have a significant effect on breast cancer risk, but the results are inconsistent. A meta-analysis based on 57 eligible studies was carried out to clarify the role of *MTHFR* gene polymorphisms in breast cancer.

**Methods and Results:**

Eligible articles were identified by searching databases including PubMed, Web of Science, EMBASE, CNKI and CBM for the period up to August 2012. Finally, a total of 57 studies were included in this meta-analysis. Crude ORs with 95% CIs were used to assess the association between the *MTHFR* polymorphisms and breast cancer risk. The pooled ORs were performed with additive model, dominant model and recessive model, respectively. Subgroup analysis was also performed by ethnicity. The statistical heterogeneity across studies was examined with χ^2^-based Q-test. A meta-analysis was performed using the Stata 12.0 software. Overall, the *677 C* allele was significantly associated with breast cancer risk (OR = 0.942, 95%CI = 0.898 to 0.988) when compared with the *677 T* allele in the additive model, and the same results were also revealed under other genetic models. Simultaneously, the *1298 A* allele was not associated with the breast cancer susceptibility when compared with the *1298 C* allele (OR = 0.993, 95%CI = 0.978 to 1.009). Furthermore, analyses under the dominant, recessive and the allele contrast model yielded similar results.

**Conclusions:**

The results of this meta-analysis suggest that *677 C>T* polymorphism in the *MTHFR* gene may contribute to breast cancer development. However, the *1298 A>C* polymorphism is not significantly associated with increased risks of breast cancer.

## Introduction

Breast cancer is currently the most common cancer among women and one of the leading causes of cancer-related death in the world [1]. The etiology of the disease is still not fully understood. Some risk factors such as familial history, age of menarche and of menopause, diet, reproductive history, high estrogen exposure as well as genetic factors may contribute to its development [2], [3]. Low-penetrance susceptibility genes combining with environmental factors have been considered as one of the important factors in the progression of cancer [4]. Recently, several common low-penetrant genes have been identified as potential breast cancer susceptibility genes, one of which is 5,10-methylenetetrahydrofolate reductase (*MTHFR*) gene. The *MTHFR* gene produces a key enzyme for intracellular folate homeostasis and metabolism, which catalyzes the conversion of 5,10-methylenetetrahydrofolate (5,10-methylene-THF) to 5-methyltetrahydrofolate (5-methylene-THF). The latter is the predominant circulating form of folate in plasma and provides the methyl group for de novo methionine synthesis through homocysteine remethylation [5].

Folates play an integral role in maintaining DNA stability by regulating DNA biosynthesis, DNA repair and DNA methylation. Low intake of folate may increase the risk of several cancers, including breast cancer [6], [7]. This reaction is essential for both purine nucleotide biosynthesis and remethylation of homocysteine to methionine used in DNA methylation [8]. Reduction of the *MTHFR* enzyme activity may increase the cancer risk through impaired DNA repair synthesis and disruption of DNA methylation. In addition, it has been suggested that breast carcinogenesis could be associated with alteration of oestrogen receptor gene methylation patterns [9] and global DNA methylation [10].

The gene encoding *MTHFR* is polymorphic located at 1p36.3 [11]. The two most common polymorphisms in the *MTHFR* gene, *677 C>T (rs1801133)* and *1298 A>C (rs1801131)*, are both associated with reduced enzyme activity [12]. The *MTHFR 677 TT* (homozygote) genotype results in 30% enzyme activity in vitro compared with the *CC* wild-type, whereas the *MTHFR 1298 CC* genotype has been found to result in 60% enzyme activity in vitro compared with the *AA* wild-type [13], [14], [15]. A series of studies have investigated the association between the two common polymorphisms of *MTHFR* gene and breast cancer susceptibility, but provided inconclusive results. Some studies found *MTHFR 677 TT* genotype is significantly associated with an increased risk of breast cancer [16], [17], [18], while no significant association in others [19], [20]. For the *1298 A>C* polymorphism, *C* allele was associated with increased risk in the studies of Ergul et al. [18] and Stevens et al. [21], while reduced risk of breast cancer was found for the heterozygous model (*A/C* vs *C/C)* by Chou et al.[22]. Hence, we conducted this systematic meta-analysis of all available studies describing the association between *MTHFR 677 C>T* and *1298 A>C* polymorphisms and the risk of breast cancer.

## Materials and Methods

### 2.1 Literature and Search Strategy

A computerized literature search was conducted for the relevant available studies published in English in PubMed, Web of Science, EMBASE, Chinese National Knowledge Infrastructure database (CNKI) and Chinese Biomedical Literature database (CBM). The literature search was updated on August 1, 2012. The search strategy identified all possible studies using combinations of the following keywords: “methylenetetrahydrofolate reductase”, “*MTHFR*”, “*MTHFR C677 T*”, “*MTHFR Ala222Val*”, “*MTHFR A1298 C*”, “*MTHFR Glu222Val*”, “folate”, “one-carbon metabolism”, “*rs1801133*”, “*rs1801131*”, “polymorphism”, “genotype”, “variant”, “breast cancer”, and “breast neoplasm”. We did not define any minimum number of subjects for the studies included in this meta-analysis. No language restrictions were imposed. The reference lists of reviewed articles, clinical trials, and meta-analyses, were also hand-searched for collecting other relevant studies. Two authors conducted all searches independently. When the same patient population was included in several publications, only the most recent or complete study was used in this meta-analysis.

### 2.2 Eligibility Criteria

The studies included in this meta-analysis had to meet the following criteria: (1) utilized platinum-based regimens for patients with pathologically proven breast cancer; (2) controls were matched with normal persons; (3) only cohort studies and case-control studies were included in this meta-analysis; (4) evaluation of the *MTHFR 677 C>T* and *1298 A>C* polymorphisms and beast cancer risk; (5) clearly described the source of cases and controls; (6) provided sample sizes, and sufficient genotyping data to calculate odds ratios (ORs) and their 95% confidence intervals (CIs).

Accordingly, the exclusion criteria were: (1) not designed as case-control or cohort studies; (2) reviews; (3) not offering the source of cases and controls and other essential information; (4) control population including malignant tumor patients; (5) duplicated publications.

### 2.3 Quality Assessment

Quality of the studies was assessed using the Newcastle–Ottawa Quality Assessment Scale for cohort studies [23],[24]. This scale is composed of eight items to assess patient selection, study comparability and outcome. The scale was recommended by the Cochrane Non-Randomized Studies Methods Working Group [25]. Two investigators performed quality assessment independently. Disagreement was resolved by consensus.

### 2.4 Data Extraction

Information was independently extracted from all eligible publications by two authors according to the inclusion and exclusion criteria listed above. Disagreement was resolved by discussion between the two authors. The following data were collected from each study: first author’s surname, year of publication, ethnicity, the numbers of cases and controls with the frequencies of *CC*, *CT* and *TT* genotypes, and the *AA*, *AC* and *CC* genotypes, respectively. Different ethnicity descents were categorized as Caucasian, Asian and Mixed population. When studies included subjects of more than one ethnicity, data were extracted separately for each ethnic group.

### 2.5 Statistical Analysis

Crude ORs with 95% CIs were used to assess the strength of association between the *MTHFR 677 C>T* and *1298 A>C* polymorphisms and breast cancer risk. For the two polymorphisms, the meta-analysis examined their associations for the allele model *C* vs *T*; homozygote (*CC* vs *TT*), recessive model (*CC* vs *CT*+*TT*) and dominant model (*CC*+*CT* vs *TT*). Subgroup analyses were stratified by ethnicity. Both fixed-effects model using the Mantel–Haenszel method and random-effects model using the DerSimonian and Laird method were used to pool the results.

Heterogeneity assumption was checked by the Chi-square-based Q-test [26]. A *P*-value greater than 0.10 for the Q-test indicates a lack of heterogeneity among studies, so the pooled OR estimate of the included studies was calculated by the fixed-effects model. Otherwise, the random-effects model was used. The significance of the pooled OR was determined by the Z-test, and *P*<0.05 was considered as statistically significant. One-way sensitivity analyses were performed to assess the stability of the results, namely, a single study in the meta-analysis was deleted each time to reflect the influence of the individual dataset on the pooled OR. An estimate of potential publication bias was carried out by the funnel plot, in which the standard error of log (OR) of each study was plotted against its log (OR). An asymmetric plot suggests a possible publication bias. Funnel plot asymmetry was assessed by the method of Egger’s linear regression test, a linear regression approach to measure funnel plot asymmetry on the natural logarithm scale of the OR. The significance of the intercept was determined by the t-test suggested by Egger (*P*<0.05 was considered representative of statistically significant publication bias) [27]. Hardy–Weinberg equilibrium in the control group was tested by the Chi-square test for goodness of fit, and a *P*-value <0.05 was considered significant. All of the calculations were performed using STATA (version 12.0; Stata Corporation, College Station, TX), using two-sided *P*-values.

## Results

### 3.1 Study Characteristics

**Figure 1:**
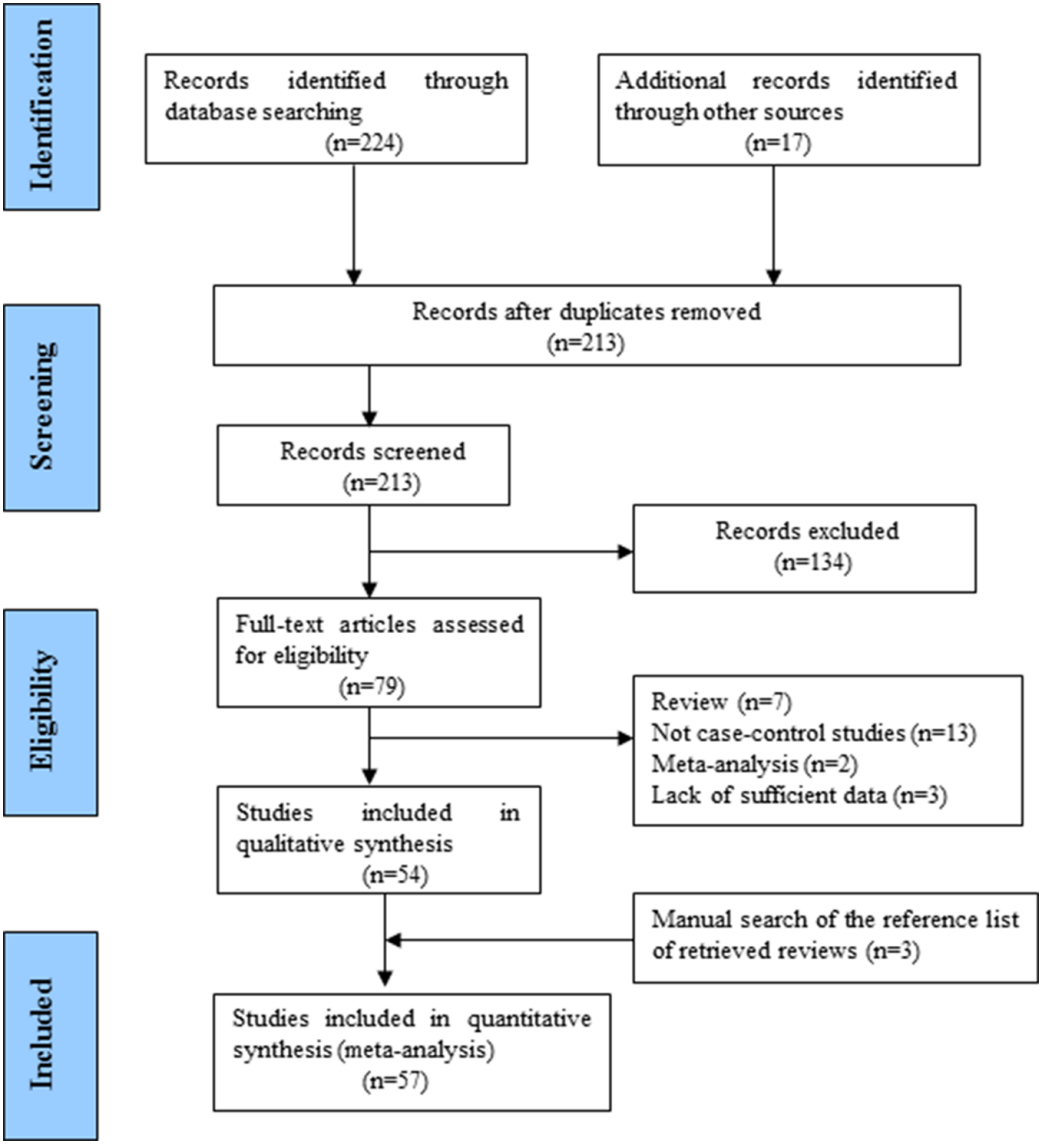
Flow diagram summarizing the search strategy for meta-analysis of MTFHR gene and breast cancer susceptibility.

Studies relevant to the searching words were retrieved originally. 57 eligible publications addressing the association between *MTHFR 677 C>T* and *1298 A>C* polymorphisms (25,877 breast cancer cases and 29,781 controls) and breast cancer risk were ultimately analyzed (**Figure 1**). All the cases were histologically confirmed. Controls were mainly healthy populations. A total of 241 articles regarding the association between *MTHFR 677 C>T* (rs1801133) and *1298 A>C* (rs1801131) polymorphisms and breast cancer were identified. After screening the duplicated articles, 28 publications were excluded. And then titles and abstracts were screened, 134 articles were excluded and 79 full-test studies were left for further evaluating. Furthermore, 25 publications were excluded because they were 7 review articles, 17 non-case control studies, 2 other meta-analysis, and 3 studies lack of sufficient data. Last, 3 studies were included in this work through manual search of the reference list of retrieved reviews. Hence, 57 publications including 57 studies for *MTHFR 677 C>T (rs1801133)* and 29 studies for *MTHFR 1298 A>C (rs1801131)* were included in this meta-analysis [28]–[73]. Simultaneously, of 57 studies for *MTHFR 677 C>T (rs1801133)* polymorphism and breast cancer susceptibility, included 28 groups of Caucasians, 20 groups of Asians, and 9 Mixed populations. While 29 studies for *MTHFR 1298 A>C (rs1801131)* polymorphism and breast cancer susceptibility, included 13 groups of Caucasians, 9 groups of Asians, and 7 Mixed populations. The distribution of genotypes in the controls of all studies was in agreement with Hardy–Weinberg equilibrium.

### 3.2 Association of the *MTHFR* Gene *677C>T* Genotype with Breast Cancer Risk

**Figure 2:**
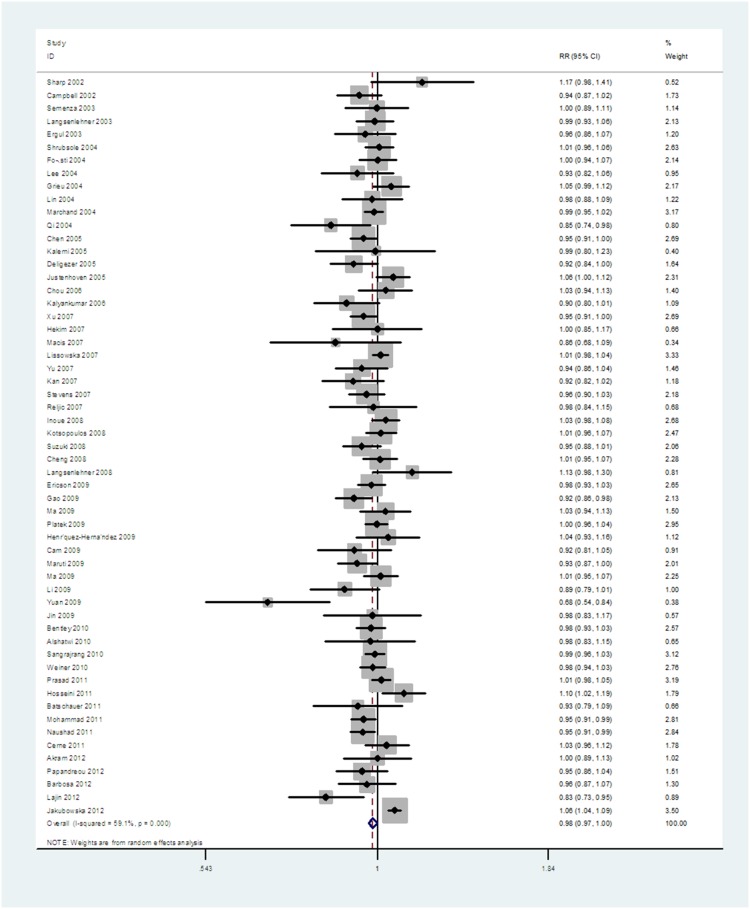
Forest plot of breast cancer susceptibility associated with *MTHFR 677 C>T* polymorphism at additive model (*C* allele vs *T* allele).

The main characteristics of these studies were listed in **Table S1**. The association between the *677 C>T* polymorphism and breast cancer risk was investigated under the additive model (allele *C* vs allele *T*). Substantial heterogeneity among the studies (*I^2^* = 59.1%, *P* = 0.0014) was found. The overall OR under a random-effects model was 0.942 (95%CI = 0.898 to 0.988), suggesting a significant association (**Figure 2**). An overall analysis under other genetic models was then performed. It also revealed a significant association under the dominant model (OR = 0.990, 95%CI = 0.982 to 0.999) and the recessive model (OR = 0.956, 95%CI = 0.923 to 0.990). Furthermore, a significant association was found under other pair-wise comparisons. The results were shown in **Table 1**. In order to analyze characteristic-homogeneous groups, subgroup analysis was carried out by ethnicity. Significant association was found under the additive genetic model.

**Table 1:** Main results of pooled odds ratios (ORs) with confidence interval (CI) in the meta-analysis.

Variables	No. of studies	*CC* vs *TT*			*CC* vs *CT*			*CT* vs *TT*		
		OR (95% CI)	Ph	P	OR (95% CI)	Ph	P	OR (95% CI)	Ph	P
Total	57	0.983(0.969 0.997)	0.000	**0.019**	0.984(0.967 1.001)	0.222	0.070	0.983(0.967 1.000)	0.002	**0.048**
Asian	20	0.770(0.633 0.938)	0.015	**0.009**	0.940(0.867 1.018)	0.244	0.130	0.845(0.730 0.977)	0.230	**0.023**
Caucasian	28	0.946(0.818 1.094)	0.006	0.456	1.010(0.952 1.072)	0.191	0.733	0.919(0.792 1.067)	0.004	0.269
Mixed	9	0.894(0.792 1.009)	0.114	0.071	0.940(0.887 0.996)	0.795	**0.036**	0.956(0.839 1.090)	0.061	0.502

Variables	No. of studies	*CC*+*CT* vs *TT* (dominant)			*CC* vs *CT*+*TT* (recessive)			*C* allele vs *T* allele		
		OR (95% CI)	Ph	P	OR (95% CI)	Ph	P	OR (95% CI)	Ph	P
Total	57	0.990(0.982 0.999)	0.000	**0.020**	0.956(0.923 0.990)	0.052	**0.011**	0.942(0.898 0.988)	0.000	**0.013**
Asian	20	0.808(0.687 0.952)	0.057	**0.011**	0.911(0.845 0.983)	0.057	**0.017**	0.877(0.801 0.960)	0.003	**0.005**
Caucasian	28	0.931(0.807 1.074)	0.002	0.329	1.004(0.950 1.062)	0.151	0.883	0.983(0.924 1.045)	0.018	0.582
Mixed	9	0.921(0.814 1.043)	0.057	0.196	0.935(0.885 0.987)	0.752	**0.016**	0.953(0.857 1.059)	0.000	0.368

P_h_: P value of Q-test for heterogeneity test.

### 3.3 Association of the *MTHFR* Gene *1298 a>C* Genotype with Breast Cancer Risk

**Figure 3:**
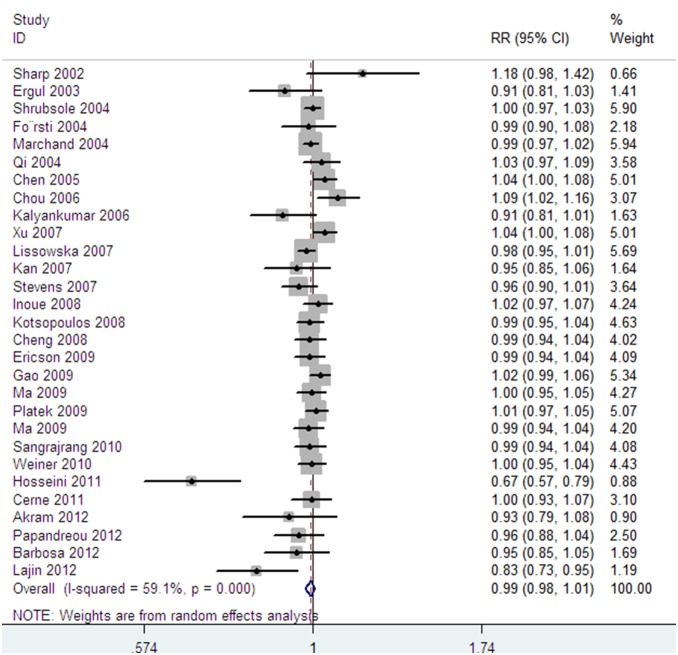
Forest plot of breast cancer susceptibility associated with *MTHFR 1298 A>C* polymorphism at additive model (*A* allele vs *C* allele).

29 studies were included in the meta-analysis to describe the association between *1298A>C* polymorphism and breast cancer risk. The main characteristics of these studies were listed in **Table S2**. Analysis of *1298 A>C* polymorphism in the *MTHFR* gene with breast cancer risk under the additive model was performed and the random model was used to assess the overall OR value. Compared with the carrier of the *C* allele, the overall OR of the *A* allele was 0.993 (95%CI = 0.978 to 1.009) (**Figure 3**). Under the recessive and the dominant models, the overall OR was 0.999 (95%CI = 0.972 to 1.027) and 0.994 (95%CI = 0.985 to 1.004), respectively. When pair-wise comparisons were made, comparing with the *C/C* genotype, the overall OR of the *A/A* genotype with breast cancer risk was 0.993 (95%CI = 0.979 to 1.008). However, compared with homozygotes of the *CC* allele, significant association of the *AC* genotype with breast cancer risk was not found and the overall OR was 0.983 (95%CI = 0.962 to 1.006). Analyses under different genetic models were shown in **Table 2**.

**Table 2:** Main results of pooled odds ratios (ORs) with confidence interval (CI) in the meta-analysis.

Variables	No. of studies	*AA* vs *CC*			*AA* vs *AC*			*AC* vs *CC*		
		OR (95% CI)	Ph	P	OR (95% CI)	Ph	P	OR (95% CI)	Ph	P
Total	29	0.993(0.979 1.008)	0.001	0.367	1.001(0.982 1.021)	0.144	0.888	0.983(0.962 1.006)	0.000	0.143
Asian	9	0.918(0.732 1.152)	0.554	0.461	1.093(0.995 1.201)	0.367	0.065	0.846(0.670 1.068)	0.552	0.159
Caucasian	13	0.781(0.629 0.972)	0.004	**0.026**	0.921(0.854 0.993)	0.634	**0.033**	0.793(0.613 1.025)	0.000	0.077
Mixed	7	1.028(0.835 1.266)	0.058	0.795	1.034(0.959 1.115)	0.232	0.379	1.012(0.843 1.215)	0.157	0.898

Variables	No. of studies	*AA*+*AC* vs *CC* (dominant)			*AA* vs *AC*+*CC*(recessive)			*A* allele vs *C* allele		
		OR (95% CI)	Ph	P	OR (95% CI)	Ph	P	OR (95% CI)	Ph	P
Total	29	0.994(0.985 1.004)	0.000	0.242	0.999(0.972 1.027)	0.021	0.955	0.993(0.978 1.009)	0.000	0.407
Asian	9	0.893(0.714 1.118)	0.554	0.324	1.074(0.981 1.176)	0.359	0.123	1.040(0.958 1.130)	0.348	0.348
Caucasian	13	0.794(0.630 1.001)	0.000	0.051	0.907(0.845 0.974)	0.221	**0.007**	0.876(0.789 0.972)	0.001	**0.013**
Mixed	7	1.025(0.848 1.238)	0.094	0.802	1.038(0.966 1.115)	0.135	0.312	1.019(0.938 1.107)	0.074	0.658

P_h_: P value of Q-test for heterogeneity test.

According to study characteristics, subgroup analysis and sensitivity analysis were performed. The results showed that the *1298A* allele in Caucasian population had significant effect on the risk of breast cancer (OR = 0.876; 95%CI = 0.789 to 0.972), whereas this effect was reversed in Asian (OR = 1.040; 95%CI = 0.958 to 1.130) and Mixed population (OR = 1.019; 95%CI = 0.938 to 1.107) (**Table 2** **)**.

### 3.4 Sensitivity Analysis

In order to compare the difference and evaluate the sensitivity of the meta-analyses, we conducted one-way sensitivity analysis to evaluate the stability of the results. The statistical significance of the results was not altered when any single study was omitted (data not shown), confirming the stability of the results.

### 3.5 Publication Bias

**Figure 4:**
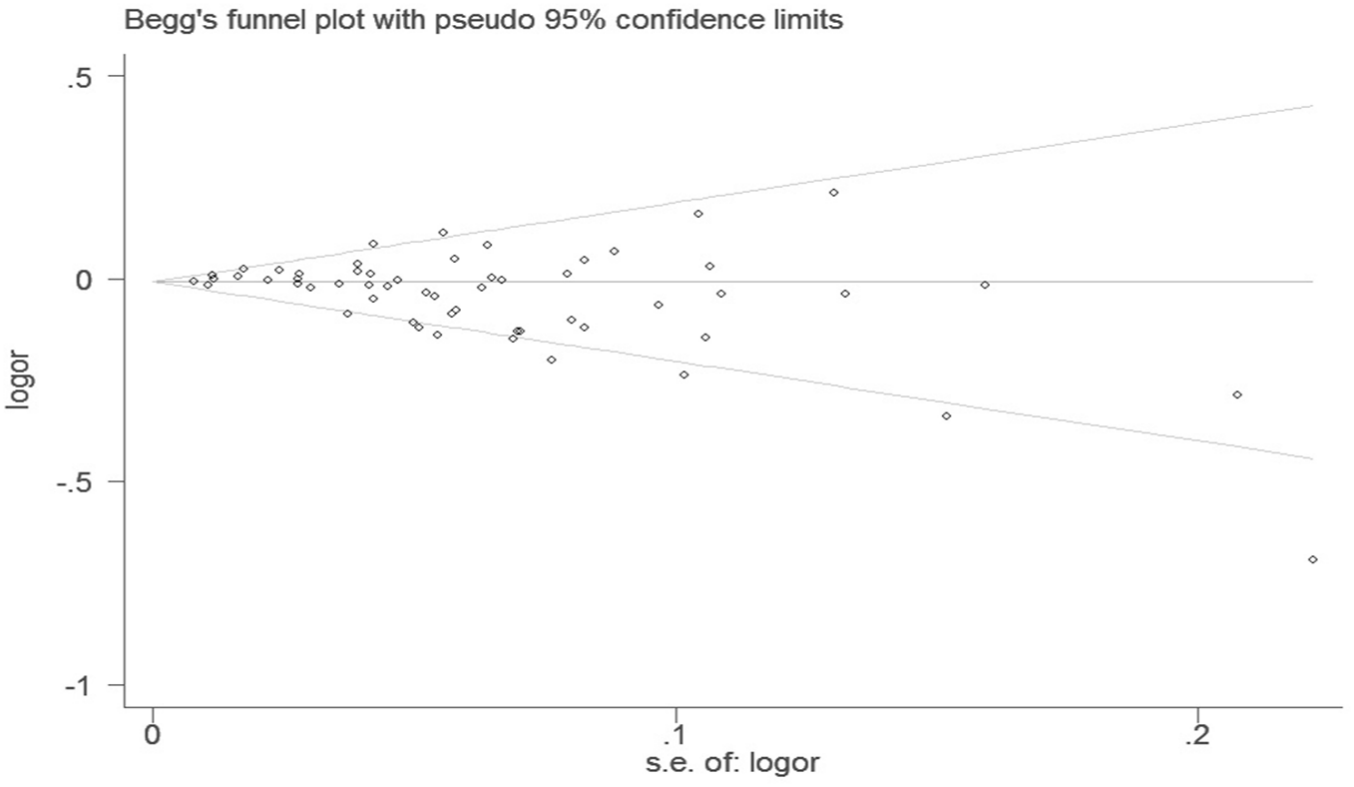
Begg’s funnel plot with pseudo 95% confidence limit under the additive genetic model of *677 C>T* genotype.

**Figure 5:**
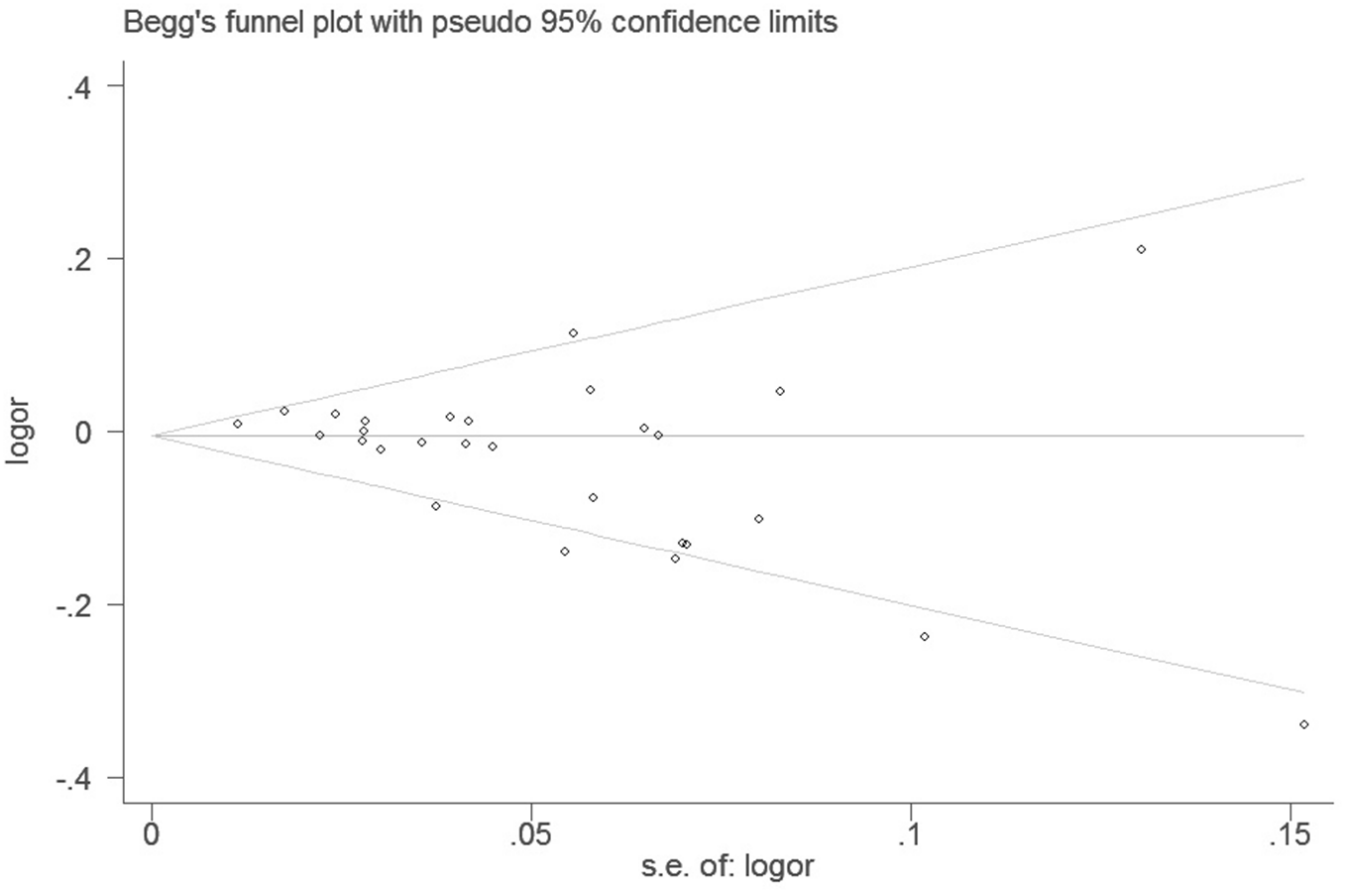
Begg’s funnel plot with pseudo 95% confidence limit under the additive genetic model of *1298 A>C* genotype.

Begg’s funnel plot and Egger’s test were performed to assess the publication bias. The shapes of the funnel plots did not reveal significant evidence of obvious asymmetry in all comparison models (Figures not show). Furthermore, Egger’s test was used to provide statistical evidence for funnel plot symmetry. The results still did not suggest any evidence of publication bias (*P* = 0.322 for *C* allele vs *T* allele, **Figure 4**; *P* = 0.066 for *A* allele vs *C* allele, **Figure 5**).

## Discussion

It is well recognized that there is individual susceptibility to the same kind of cancer even with the same environmental exposure. Environmental factors and gene genotypes involved in carcinogenesis may account for this difference. Therefore, genetic susceptibility to cancer has been a research focus in scientific community. Genetic epidemiologic studies of single nucleotide polymorphism can provide the relationships between candidate genes and cancer risk. However, individual studies on the relationship between *MTHFR 677 C>T* and *1298 A>C* polymorphisms and cancer risk generated inconsistent results partly because of the small sample size. Meta-analysis is a method that can solve the problem caused by low statistical power in single study to draw a more robust conclusion. Our present study, including 57 published cohort and/or case-control studies, estimated the potential role of *MTHFR 677 C>T* and *1298 A>C* polymorphisms in breast cancer development.

In the meta-analysis of the *677 C>T* polymorphism, a total of 57 studies involving 55,658 subjects, significant association with breast cancer risk was detected in overall comparisons under all genetic models. Results from studies with small sample size or deviating HWE are inconsistent. One explanation may be that small sample size and deviation form HWE may have biased the results. Study characteristics, such as mean age of cases, status of premenopausal and postmenopausal, genotyping method, study design, source of controls and ethnicity, showed some differences in the included studies. But most of them were not responsible for heterogeneity by subgroup analysis and sensitivity analysis.

Another variant of the *MTHFR* gene, the *1298 A>C* polymorphism, which is present in 27,141 subjects and 29 studies, showed that the *A* allele was not significantly associated with increased breast cancer risk when compared with the *C* allele under additive, recessive model and the *AA* vs *CC* genetic model. In the subgroup analysis, *A* allele was not associated the risk of breast cancer in Caucasian populations.

Several potential limitations of this meta-analysis should be considered. First, this meta-analysis mostly focused on papers published in English and Chinese. Second, not all the control subjects were age and sex matched to cases, which may introduce heterogeneity in this meta-analysis. Third, subgroup analysis was only performed according to ethnicity, due to the unavailability of the data on the status of menopausal and conditions on folate intake. Forth, the insufficient information in the included studies did not allow further analysis of the joint effects of the two polymorphisms. However, the advantages of this meta-analysis were also obvious. The large sample size of this study confirmed the reliability of our results. Second, the potential sources of heterogeneity in the meta-analysis were assessed. Third, the associations between the *MTHFR 677 C>T* and *1298 A>C* polymorphisms and breast cancer risk were evaluated under different genetic models**.**

In conclusion, although the meta-analysis provides evidence that the *MTHFR 1298 A>C* polymorphism is not significantly associated with increased risk of breast cancer, a significant association was found between the *MTHFR 677 C>T* polymorphism and breast cancer risk, especially in Asian populations. Well-designed and large studies are needed to further investigate the association of these polymorphisms with breast cancer susceptibility.

## Supporting Information

Table S1 **The main characteristics of these studies included in this meta-analysis and the distribution of MTHFR gene 677C>T genotypes and alleles among cases and controls.**
(DOCX)

Table S2 **The main characteristics of these studies included in this meta-analysis and the distribution of MTHFR gene 1298A>C genotypes and alleles among cases and controls.**
(DOCX)

Checklist S1 **PRISMA Checklist.**
(DOC)
