# Supplementary material for: Association of 677 C>T (rs1801133) and 1298 A>C (rs1801131) Polymorphisms in the MTHFR Gene and Breast Cancer Susceptibility: A Meta-Analysis Based on 57 Individual Studies
Source: PLoS One. 2014 Jun 19;9(6):e71290. doi: 10.1371/journal.pone.0071290 (PMC4063741; doi:10.1371/journal.pone.0071290)
Supplement: Table S1 — The main characteristics of these studies included in this meta-analysis and the distribution of MTHFR gene 677C>T genotypes and alleles among cases and controls. (DOCX) [file pone.0071290.s001.docx]

**Table S1.** The main characteristics of these studies included in this meta-analysis and the distribution of MTHFR gene 677C>T genotypes and alleles among cases and controls.

| **First author**  **[Inference]** | **Year** | **Ethnicity** | **Source of controls** | **Cases** | | | **Controls** | | |
| --- | --- | --- | --- | --- | --- | --- | --- | --- | --- |
|  |  |  |  | **CC** | **CT** | **TT** | **CC** | **CT** | **TT** |
| Sharp [28] | 2002 | Caucasian | PB | 30 | 19 | 5 | 25 | 21 | 11 |
| Campbell [29] | 2002 | Caucasian | HB | 140 | 162 | 33 | 118 | 92 | 23 |
| Semenza [30] | 2003 | Caucasian | HB | 42 | 58 | 5 | 112 | 111 | 24 |
| Langsenlehner [31] | 2003 | Caucasian | PB | 208 | 222 | 64 | 215 | 215 | 65 |
| Ergul [18] | 2003 | Caucasian | HB | 60 | 41 | 17 | 94 | 87 | 12 |
| Shrubsole [32] | 2004 | Asian | PB | 374 | 555 | 183 | 387 | 577 | 196 |
| Fo¨rsti [33] | 2004 | Caucasian | NA | 134 | 81 | 8 | 181 | 104 | 13 |
| Lee [34] | 2004 | Asian | HB | 58 | 96 | 32 | 50 | 80 | 17 |
| Grieu [35] | 2004 | Caucasian | PB | 166 | 141 | 27 | 242 | 259 | 50 |
| Lin [36] | 2004 | Asian | PB | 43 | 38 | 7 | 173 | 145 | 24 |
| Le Marchand [37] | 2004 | Mixed | PB | 573 | 479 | 137 | 1211 | 920 | 283 |
| Qi [38] | 2004 | Asian | PB | 42 | 104 | 71 | 59 | 105 | 54 |
| Chen [19] | 2005 | Mixed | PB | 398 | 476 | 189 | 440 | 509 | 155 |
| Kalemi [39] | 2005 | Caucasian | NA | 19 | 16 | 7 | 23 | 20 | 8 |
| Deligezer [16] | 2005 | Caucasian | NA | 98 | 68 | 23 | 128 | 83 | 12 |
| Justenhoven [17] | 2005 | Caucasian | PB | 249 | 247 | 61 | 261 | 279 | 93 |
| Chou [22] | 2006 | Asian | HB | 73 | 51 | 18 | 132 | 120 | 33 |
| Kalyankumar [40] | 2006 | Caucasian | PB | 45 | 37 | 6 | 61 | 31 | 3 |
| Lissowska [41] | 2007 | Caucasian | PB | 982 | 815 | 177 | 1132 | 915 | 235 |
| Xu [42] | 2007 | Mixed | PB | 398 | 476 | 189 | 440 | 509 | 155 |
| Hekim [43] | 2007 | Caucasian | NA | 22 | 16 | 2 | 38 | 26 | 4 |
| Macis [44] | 2007 | Caucasian | PB | 14 | 20 | 12 | 28 | 41 | 11 |
| Yu [45] | 2007 | Asian | PB | 56 | 54 | 9 | 225 | 170 | 25 |
| Kan [46] | 2007 | Asian | PB | 74 | 29 | 22 | 65 | 29 | 9 |
| Stevens [21] | 2007 | Mixed | PB | 208 | 224 | 62 | 236 | 193 | 65 |
| Reljic [47] | 2007 | Caucasian | PB | 40 | 44 | 9 | 27 | 34 | 4 |
| Inoue [48] | 2008 | Asian | PB | 239 | 120 | 21 | 393 | 226 | 43 |
| Kotsopoulos [49] | 2008 | Caucasian | HB | 383 | 421 | 140 | 252 | 341 | 87 |
| Suzuki [50] | 2008 | Asian | HB | 150 | 220 | 84 | 338 | 425 | 146 |
| Cheng [51] | 2008 | Asian | HB | 185 | 133 | 31 | 268 | 221 | 41 |
| Langsenlehner [52] | 2008 | Caucasian | NA | 51 | 43 | 11 | 40 | 48 | 17 |
| Ericson [53] | 2009 | Caucasian | PB | 255 | 235 | 50 | 531 | 452 | 91 |
| Gao [54] | 2009 | Asian | PB | 202 | 305 | 117 | 235 | 301 | 88 |
| Ma [55] | 2009 | Asian | HB | 124 | 183 | 81 | 115 | 188 | 84 |
| Platek [20] | 2009 | Mixed | PB | 429 | 446 | 119 | 788 | 795 | 219 |
| Henrı´quez-Herna´ndez [56] | 2009 | Caucasian | PB | 52 | 65 | 18 | 107 | 138 | 47 |
| Cam [57] | 2009 | Caucasian | NA | 48 | 49 | 13 | 47 | 42 | 6 |
| Maruti [58] | 2009 | Mixed | PB | 133 | 139 | 46 | 301 | 284 | 62 |
| Ma [59] | 2009 | Mixed | HB | 225 | 188 | 45 | 222 | 187 | 49 |
| Li [60] | 2009 | Asian | PB | 38 | 17 | 10 | 90 | 50 | 3 |
| Yuan [57] | 2009 | Asian | HB | 16 | 35 | 29 | 32 | 35 | 13 |
| Jin [58] | 2009 | Asian | NA | 18 | 20 | 3 | 49 | 41 | 10 |
| Bentley [59] | 2010 | Caucasian | HB | 346 | 402 | 191 | 429 | 529 | 205 |
| Alshatwi [60] | 2010 | Asian | PB | 34 | 50 | 16 | 36 | 49 | 15 |
| Sangrajrang [61] | 2010 | Asian | HB | 410 | 144 | 9 | 366 | 110 | 11 |
| Weiner [62] | 2010 | Caucasian | HB | 399 | 364 | 74 | 386 | 326 | 66 |
| Prasad [63] | 2011 | Asian | HB | 124 | 5 | 1 | 116 | 8 | 1 |
| Hosseini [64] | 2011 | Caucasian | HB | 168 | 84 | 42 | 150 | 90 | 60 |
| Batschauer [65] | 2011 | Caucasian | HB | 27 | 34 | 7 | 42 | 34 | 9 |
| Mohammad [66] | 2011 | Asian | HB | 168 | 53 | 1 | 198 | 37 | 0 |
| Naushad [67] | 2011 | Asian | PB | 185 | 56 | 3 | 205 | 39 | 0 |
| Cerne [68] | 2011 | Caucasian | PB | 222 | 238 | 62 | 108 | 124 | 37 |
| Akram [69] | 2012 | Caucasian | HB | 65 | 25 | 20 | 55 | 45 | 10 |
| Papandreou [70] | 2012 | Caucasian | HB | 105 | 150 | 45 | 99 | 161 | 23 |
| Barbosa [71] | 2012 | Mixed | PB | 76 | 83 | 17 | 87 | 70 | 19 |
| Lajin [72] | 2012 | Caucasian | HB | 44 | 52 | 23 | 65 | 48 | 13 |
| Jakubowska [73] | 2012 | Mixed | HB | 2032 | 2166 | 580 | 1447 | 1481 | 422 |
| PB: Population-Based Study; HB：Hospital-Based Study. NA: Not Avaliable | | | | | | | | | |
