# Supplementary material for: Association of 677 C>T (rs1801133) and 1298 A>C (rs1801131) Polymorphisms in the MTHFR Gene and Breast Cancer Susceptibility: A Meta-Analysis Based on 57 Individual Studies
Source: PLoS One. 2014 Jun 19;9(6):e71290. doi: 10.1371/journal.pone.0071290 (PMC4063741; doi:10.1371/journal.pone.0071290)
Supplement: Table S2 — The main characteristics of these studies included in this meta-analysis and the distribution of MTHFR gene 1298A>C genotypes and alleles among cases and controls. (DOCX) [file pone.0071290.s002.docx]

**Table S2.** The main characteristics of these studies included in this meta-analysis and the distribution of MTHFR gene 1298A>C genotypes and alleles among cases and controls**.**

| First author  [Inference] | Year | Ethnicity | Source of controls | Cases | | | Controls | | |
| --- | --- | --- | --- | --- | --- | --- | --- | --- | --- |
|  |  |  |  | AA | AC | CC | AA | AC | CC |
| Sharp [28] | 2002 | Caucasian | PB | 27 | 25 | 3 | 24 | 25 | 11 |
| Ergul [18] | 2003 | Caucasian | HB | 50 | 48 | 20 | 90 | 85 | 18 |
| Shrubsole [32] | 2004 | Asian | PB | 768 | 311 | 42 | 824 | 344 | 40 |
| Fo¨rsti [33] | 2004 | Caucasian | NA | 94 | 102 | 27 | 133 | 127 | 38 |
| Marchand [37] | 2004 | Mixed | PB | 741 | 372 | 77 | 1493 | 801 | 120 |
| Qi [38] | 2004 | Asian | PB | 155 | 58 | 4 | 144 | 71 | 3 |
| Chen [19] | 2005 | Mixed | PB | 558 | 417 | 87 | 536 | 457 | 110 |
| Chou [22] | 2006 | Asian | HB | 104 | 30 | 8 | 172 | 95 | 18 |
| Kalyankumar [40] | 2006 | Caucasian | PB | 49 | 33 | 6 | 65 | 26 | 4 |
| Lissowska [41] | 2007 | Caucasian | PB | 892 | 874 | 220 | 1086 | 941 | 251 |
| Xu [42] | 2007 | Mixed | PB | 558 | 417 | 87 | 536 | 457 | 110 |
| Kan [46] | 2007 | Asian | PB | 70 | 41 | 14 | 61 | 32 | 8 |
| Stevens [21] | 2007 | Mixed | PB | 224 | 228 | 42 | 252 | 201 | 40 |
| Inoue [48] | 2008 | Asian | PB | 225 | 139 | 16 | 387 | 234 | 41 |
| Kotsopoulos [49] | 2008 | Caucasian | HB | 466 | 390 | 85 | 398 | 309 | 73 |
| Cheng [51] | 2008 | Asian | HB | 207 | 125 | 19 | 310 | 207 | 17 |
| Ericson [53] | 2009 | Caucasian | PB | 242 | 242 | 57 | 487 | 480 | 105 |
| Gao [54] | 2009 | Asian | PB | 446 | 165 | 9 | 425 | 188 | 11 |
| Ma [59] | 2009 | Asian | HB | 254 | 119 | 15 | 256 | 116 | 15 |
| Platek [20] | 2009 | Mixed | PB | 443 | 402 | 83 | 842 | 758 | 181 |
| Ma [59] | 2009 | Mixed | HB | 269 | 168 | 21 | 279 | 157 | 22 |
| Sangrajrang [61] | 2010 | Asian | HB | 302 | 223 | 38 | 258 | 206 | 23 |
| Weiner [62] | 2010 | Caucasian | HB | 398 | 353 | 80 | 379 | 330 | 76 |
| Hosseini [64] | 2011 | Caucasian | HB | 36 | 96 | 162 | 60 | 135 | 105 |
| Cerne [687] | 2011 | Caucasian | PB | 258 | 219 | 47 | 131 | 117 | 21 |
| Akram [69] | 2012 | Caucasian | HB | 35 | 55 | 20 | 30 | 75 | 5 |
| Papandreou [70] | 2012 | Caucasian | HB | 129 | 135 | 36 | 136 | 116 | 31 |
| Barbosa [71] | 2012 | Mixed | PB | 68 | 80 | 17 | 72 | 84 | 9 |
| Lajin [72] | 2012 | Caucasian | HB | 44 | 52 | 23 | 65 | 48 | 13 |
| PB: Population-Based Study; HB：Hospital-Based Study. NA: Not Avalible. | | | | | | | | | |
